# A Selective Pharmacophore Model for β_2_-Adrenoceptor Agonists

**DOI:** 10.3390/molecules14114486

**Published:** 2009-11-06

**Authors:** Rui-Juan Xing, Jian Wang, Li Pan, Mao-Sheng Cheng

**Affiliations:** Key Laboratory of Structure-Based Drugs Design & Discovery of Ministry of Education, School of Pharmaceutical Engineering, Shenyang Pharmaceutical University, Shenyang 110016, China; E-Mails: ruijuanxing@163.com (R.-J.X.); wjmed@163.com (J.W.); panli65@yahoo.com.cn (L.P.)

**Keywords:** β_2_-adrenoceptor agonists, selectivity, pharmacophore, molecular field-based similarity

## Abstract

β_2_-Adrenoceptor selectivity is an important consideration in drug design in order to minimize the possibility of side effects. A selective pharmacophore model was developed based on a series of selective β_2_-adrenoceptor agonists. The best pharmacophore hypothesis consisted of five chemical features (one hydrogen-bond acceptor, one hydrogen-bond donor, two ring aromatic and one positive ionizable feature). The result was nearly in accordance with the reported interactions between the β_2_-adrenoceptor and agonists, and it shared enough similar features with the result of field point patterns by FieldTemplater, which mainly validated the pharmacophore model. Moreover, the pharmacophore could predict the selectivity over the β_1_-adrenoceptor. These results might provide guidance for the rational design of novel potent and selective β_2_-adrenoceptor agonists.

## Introduction

β-Adrenoceptors are among the most thoroughly studied members of the G protein-coupled receptors (GPCRs), the largest signaling family of the human genome. β-Adrenergic receptors have been subdivided into at least three distinct groups: β_1_, β_2_ and β_3_ [1], found predominately in cardiac muscle, airway smooth muscle and adipose tissue, respectively. Stimulation of β_2_-adrenoreceptor induces relaxation of the bronchiolar, improvement of mucociliary clearance and inhibition of extravasation of plasma proteins [2]. Therefore, β_2_-agonists are the first-line drugs for treatment of asthma and chronic obstructive pulmonary disease (COPD).

To avoid unwanted β-adrencoptor mediated effects like tachycardia, hypokalemia or muscle tremors, the sub-type specificity of the human β-receptors is considered as one of the main aspects in development of β_2_-agonists [3]. During the past decades, a lot of selective β_2_-agonists have been well developed [4]. To simplify the management of asthma and COPD, there has been a renewed interest in the development of β_2_-adrenoceptor agonists with long duration of action [5]. The key interactions between the β_2_-adrenoceptor and agonists were identified by a combination of site-directed mutagenesis and molecular modeling. The protonated nitrogen atom formed an ion pair with the carboxylate side chain of Asp113 in transmembrane (TM) 3 [6]. The catechol mimic interacted with Ser203, Ser204 and Ser207 on TM5 [7,8], and the benzylic alcohol bound to the chirally discriminating Asn293 on TM6 [9]. Besides, Tyr308 on TM7 was identified to interact with the amino-substituents of formoterol and salmeterol [10]. 

Until 2007, no X-ray structures of the human β_2_-adrenoreceptor had been reported [11,12,13]. Since then, many researchers have devoted themselves to the discovery of novel chemical classes targeting the β_2_-adrenergic receptor [14,15,16,17,18]. In the case of β_2_-agonists, biophysical studies revealed that a single small ligand could induce at least two kinetically and functionally distinct conformational states [19]. For an inactive state, the crystal structure of β_2_-adrenoceptor bound to the inverse agonist carazolol was unsuitable to directly find the pharmacophoric features for the binding of agonists in the absence of crystal packing effects.

The increasing knowledge of the β_2_-adrenoceptor structure, activity and the mode of interaction between receptor and agonists, is giving momentum to the development of computational models, such as a pharmacophore. Pharmacophore modeling has been one of the important and successful ligand-based approaches for new drug discovery in the last few years [20,21,22]. A pharmacophore hypothesis collects common features distributed in three-dimensional space representing groups in a molecule that participate in important interactions between drug and active site [23]. 

Since there is no literature concerning pharmacophore modeling for β_2_-agonists, we collected several selective β_2_-agonists with long duration of action to construct selective pharmacophore model to shed more light on the chemical features which might contribute to the β_2_-adrenogic receptor agonistic activity.

## Results and Discussion

The HipHop module within Catalyst is the common features hypotheses generation program which is widely used in ligand-based approaches to rational drug design [24]. HipHop attempts to derive a pharmacophore based on features that are common to active molecules. The constructed 3D pharmacophore model can be used for identiﬁcation of original lead compounds from a database [25,26]. 

**Figure 1:**
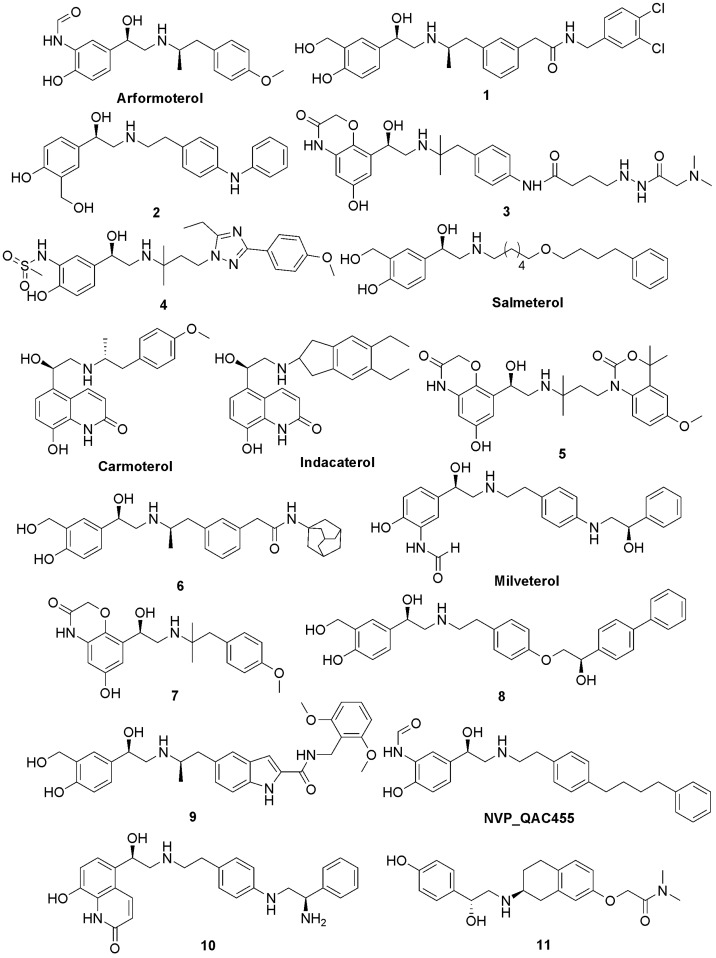
Chemical structures of the selective β_2_-agonists used to construct the HipHop pharmacophore hypotheses.

Based on the published literature, we selected a series of potent and selective β_2_-agonists (Figure 1) to generate a pharmacophore model. These compounds were taken from different literature sources [10,27,28,29,30,31,32,33,34,35,36,37,38,39,40], and the activity values were measured in various systems. In addition, due to limited structural diversity, qualitative HipHop pharmacophore modeling was performed based on the collected β_2_-agonists. 

**Figure 2:**
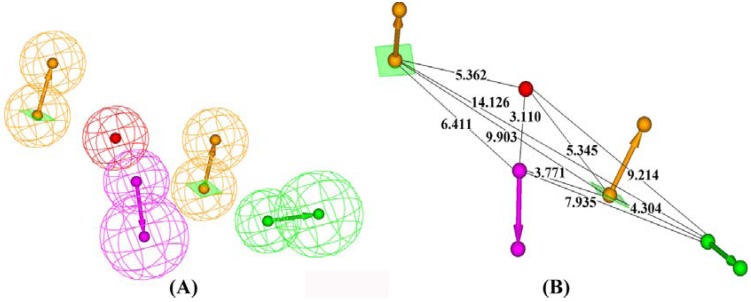
Pharmacophore model for β_2_-adrenoreceptor agonists generated by HipHop. (A) The best HipHop model Hypol_I. (B) 3D spatial relationship and geometric parameters of Hypol_I. These features are portrayed as mashed spheres, color-coded as follows: green, hydrogen-bond acceptor, magenta, hydrogen-bond donor, orange, aromatic ring, red, positive ionizable feature.

Finally ten pharmacophore hypotheses were automatically generated with alignment scores ranging from 232.507 to 240.129. (Table 1) The best hypothesis (referred to as Hypo1_I) was found to be associated with a five point pharmacophore containing one hydrogen-bond acceptor (A), one hydrogen-bond donor (D), two ring aromatic (R) and one positive ionizable feature (P). It was denoted as RRPDA and is depicted in Figure 2 showing the 3D space and distance between pharmacophoric sites. 

**Table 1:** The results of the top 10 hypotheses for β_2_-adrenoreceptor agonists generated by the HipHop program.

Hypotheses	Features	Rank	Direct Hit	Partial Hit	Max Fit
**1**	RRPDA	240.129	11111111111111111	00000000000000000	5
**2**	RRPDA	240.129	11111111111111111	00000000000000000	5
**3**	RRPDA	240.129	11111111111111111	00000000000000000	5
**4**	RRPAA	236.729	11111111111111111	00000000000000000	5
**5**	RRPAA	236.729	11111111111111111	00000000000000000	5
**6**	RRPAA	236.729	11111111111111111	00000000000000000	5
**7**	RRPDA	232.579	11111111111111111	00000000000000000	5
**8**	RRPDA	232.579	11111111111111111	00000000000000000	5
**9**	RRPAA	232.507	11111111111111111	00000000000000000	5
**10**	RRPAA	232.507	11111111111111111	00000000000000000	5

The alignment of the most potent compound arformoterol onto Hypo1_I is shown in 3A. It was revealed that one hydrogen-bond acceptor and one hydrogen-bond donor matched the 3-formamido group on the benzene ring and the benzylic alcohol, respectively. One positive ionizable feature was at the protonated nitrogen atom and the two ring aromatic features are located in the mother ring and amino-substituents. All the pharmacophores were located in the proximity, which was in good agreement with the key interactions between the β_2_-adrenoceptor and agonists.

**Table 2:** The information about the alignment of β_2_-adrenoreceptor agonists onto Hypo1_I.

Index	Name	Principal	MaxOmitFeat	FitValue
**1**	**Arformoterol**	2	0	5
**2**	**NVP_QAC455**	1	0	4.669
**3**	**4**	1	0	4.371
**4**	**6**	1	0	4.358
**5**	**Carmoterol**	1	0	4.322
**6**	**2**	1	0	4.172
**7**	**8**	1	0	4.092
**8**	**10**	1	0	3.886
**9**	**5**	1	0	3.886
**10**	**1**	1	0	3.881
**11**	**Indacaterol**	1	0	3.87
**12**	**9**	1	0	3.847
**13**	**Milveterol**	1	0	3.735
**14**	**7**	1	0	3.672
**15**	**3**	1	0	3.63
**16**	**11**	1	0	3.402
**17**	**Salmeterol**	1	0	2.66

**Figure 3:**
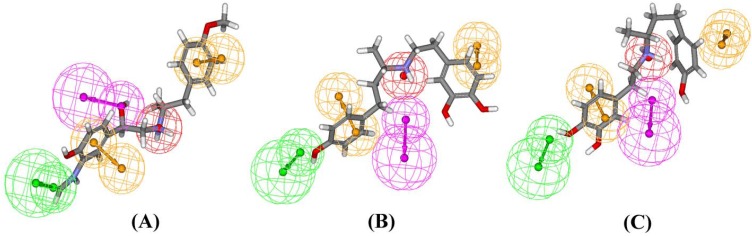
Hypol_I aligned with arformoterol (A), (*S*)-dobutamine (B) and (*R*)-dobutamine (C).

**Figure 4:**
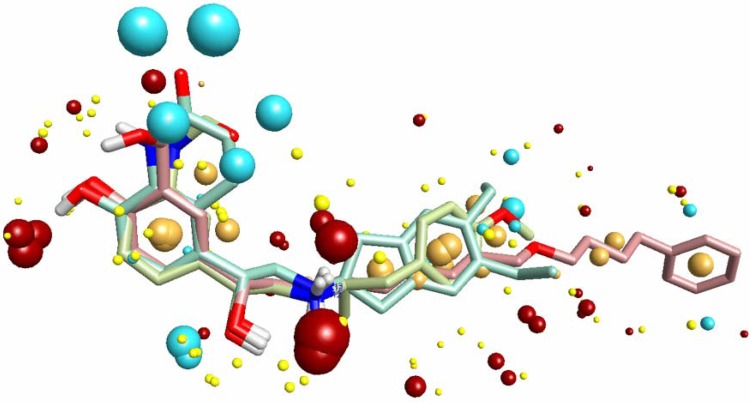
Field point patterns of the three conformers of arformoterol, salmeterol and indacaterol in Template T1. Field points are color coded as follows: negative charge, blue; positive charge, red; van der Waal’s surface, yellow; hydrophobes, orange.

Molecular field-based similarity analysis with the FieldTemplater software provided the necessary three-dimensional molecular field properties of the β_2_-adrenoceptor agonists. FieldTemplater took three β_2_-adrenoceptor agonists (arformoterol, salmeterol and indacaterol), optimally aligned their conformer fields and yielded a series of templates which were ranked according to an incorporated score. The top-ranking multi structural template (T1) from FieldTemplater is shown in Figure 4. Four types of 3D molecular field descriptors were produced for describing electrostatic (negative and positive), van der Waals and hydrophobic properties of the β_2_-adrenoceptor agonists. As shown in the field point patterns (large points indicating strong interactions), it was abundant in hydrophobic fields along the amino-substituents, positive ionic fields surrounding protonated nitrogen atoms (strong interactions) and *para*-positions of aromatic ring and negative fields near the *meta*-positions of aromatic rings (strong interactions) and surrounding the benzylic hydroxyl group. This pharmacophore shared enough similar features with Hypol_I and they were almost consistent. The results reflected the validity of the pharmacophore model Hypol_I.

**Figure 5:**
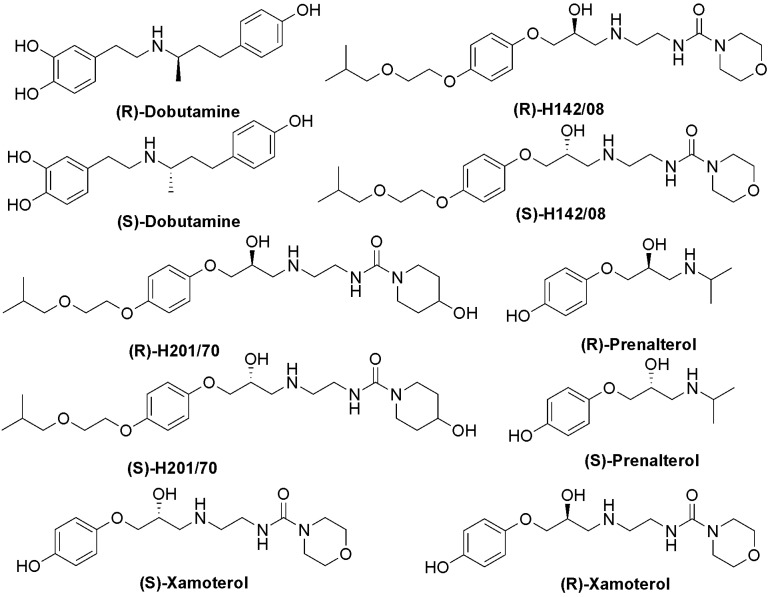
Chemical structures of selective β_1_-agonists used to map with Hypol_I.

To test the selectivity of pharmacophore over β_1_-adrenoceptor, several selective β_1_-adrenoceptor agonists (Figure 5) were collected and aligned with the Hypol_I model. As a result, no β_1_-adrenoceptor agonists could map the features using the Hypol_I model, except for dobutamine. The fit values for (*R*)-dobutamine and (*S*)-dobutamine were only 0.803 and 1.528, respectively. The alignment of (*R*)-dobutamine and (*S*)-dobutamine onto the pharmacophore is shown in Figure 3B, Figure 3C. It was found that the mapping could not match the hydrogen-bond donor and one ring aromatic feature encoded in Hypol_I. According to this partial mapping, it suggested this hydrogen-bond donor and one ring aromatic feature on the *N*-substituent might be the key areas for the differentiation of β_2_-agonists from β_1_-agonists. It has been reported that the substituent on the amino portion was important for subtype selective agonist binding [41], which further validated our phamacophore. In summary, it may be concluded that our pharmacophore model for β_2_-agonists could predict the sub-type specificity of the human β-receptors. 

Molecular docking has established itself as a valuable *in silico* technique alongside traditional high-throughput screening (HTS) for discovering new active compounds in the pharmaceutical industry. However, virtual screening of large databases via docking is expensive in terms of CPU-time, especially for GPCRs where the protein conformation changes upon binding with different ligands (ligand-induced fit). Therefore, pharmacophore models can be used as a first-screen before docking studies. Our pharmacophore model for β_2_-adrenergic receptor agonists can be used for virtual screening of new potent and selective candidates targeting the β_2_-adrenergic receptor.

## Experimental

### Pharmacophore modeling with Discovery Studio

A common-features pharmacophore model was derived with the HipHop module of Catalyst. All molecules were built in 2D Visualizer using the Discovery Studio software package and the nitrogen atom of aliphatic amine in each structure was protonated. Arformoterol was considered as ‘reference compound’ specifying a ‘Principal’ value of 2 and a ‘MaxOmitFeat’ value of 0, meaning its structure and conformation would have the strongest influence in the model building phase. The ‘Principal’ value and ‘MaxOmitFeat’ value for the remaining compounds were set to 1 and 0, respectively. Diverse conformational models for each compound were generated using the ‘best conformational analysis’ method and an energy threshold of 20 kcal/mol above the global energy minimum for conformation searching. The maximum number of conformers for each molecule was specified as 250 to ensure maximum coverage of the conformational space. Due to the basic structures of the compounds, five kinds of features including hydrogen-bond acceptor (A), hydrogen-bond donor (D), hydrophobic group (H), positive ionizable (P) and ring aromatic (R) features were selected to initiate the pharmacophore hypotheses generation process. 

### Molecular field-based similarity analysis with FieldTemplater

Using the XEDEX software, a conformational search was performed with the XED force field. Three β_2_-adrenoceptor agonists (arformoterol, salmeterol and indacaterol) were submitted to the FieldTemplater software for generation of putative bioactive field template [42,43]. FieldTemplater overlaid the field patterns of all conformers of these compounds to find a single common field pattern assumed to reflect the binding requirements for the β_2_-adrenoreceptor agonists.

### Ligand pharmacophore mapping with Discovery Studio

The β_1_-agonists were built in 2D Visualizer using Discovery Studio software package and the nitrogen atom of aliphatic amine in each structure was protonated. Diverse conformational models for each compound were generated using the ‘best conformational analysis’ method and an energy threshold of 20 kcal/mol above the global energy minimum for conformation searching. A flexible fitting method was adopted to map these β_1_-agonists with Hypol_I.

## Conclusions

In summary, we have presented the first study using a ligand-based computational approach to generate specific 3D pharmacophore hypotheses for the β_2_ aderonergic receptor from its selective agonists. The best hypothesis with a five point pharmacophore was consistent with the interactions between the β_2_-adrenoceptor and agonists, and it was further validated by molecular field-based similarity analysis with FieldTemplater. Moreover, the best pharmacophore hypothesis could perfectly differentiate β_2_-agonists from β_1_-agonists, so this pharmacophore model may be considered a valuable tool to predict agonist activity and identify diverse structures with desired biological activity and selectivity by 3D virtual screening. 
